# Can We Create a Circular Pharmaceutical Supply Chain (CPSC) to Reduce Medicines Waste?

**DOI:** 10.3390/pharmacy8040221

**Published:** 2020-11-18

**Authors:** Abdullah Alshemari, Liz Breen, Gemma Quinn, Uthayasankar Sivarajah

**Affiliations:** 1 School of Management, Faculty of Management, Law and Social Sciences, University of Bradford, Bradford BD7 1DP, UK; U.Sivarajah@bradford.ac.uk; 2 School of Pharmacy and Medical Sciences, University of Bradford, Bradford BD7 1DP, UK; L.Breen@bradford.ac.uk (L.B.); G.Quinn@bradford.ac.uk (G.Q.)

**Keywords:** waste, reuse, reduce, pharmaceutical, medicines, hospital, circular economy

## Abstract

Background: The increase in pharmaceutical waste medicines is a global phenomenon and financial burden. The Circular Economy, as a philosophy within the pharmaceutical supply chain, aims to promote waste reduction, maximise medicines value, and enable sustainability within this supply chain (increasing circularity). Circularity strategies for pharmaceuticals are not currently implemented in many countries, due to quality and safety barriers. The aim of this study was to determine whether the application of circular economy principles can minimise pharmaceutical waste and support sustainability in the pharmaceutical supply chain; Methods: a detailed narrative literature review was conducted in order to examine pharmaceutical waste creation, management, disposal, and the application of circular economy principles; Results: the literature scrutinised revealed that pharmaceutical waste is created by multiple routes, each of which need to be addressed by pharmacists and healthcare bodies through the Circular Economy 9R principles. These principles act as a binding mechanism for disparate waste management initiatives. Medicines, or elements of a pharmaceutical product, can be better managed to reduce waste, cost, and reduce negative environmental impacts through unsafe disposal. Conclusions: the study findings outline a Circular Pharmaceutical Supply Chain and suggests that it should be considered and tested as a sustainable supply chain proposition.

## 1. Introduction

The World Health Organization (WHO) defines pharmaceutical waste as undesirable pharmaceuticals, including expired, unused, spilled, and infected pharmaceutical products, medications, vaccines, and sera that are not required and should be disposed of appropriately [1]. The volume of pharmaceutical waste has increased primarily due to growth in the number of patients and prescriptions and the use and overproduction of medicines. The increase in unused, expired, and misplaced medicines contributes to medicine shortages, higher percentages of pharmaceuticals waste, and increased medicine disposal costs, and it is a growing concern globally requiring a systemic approach to its resolution [2].

According to the Pharmaceutical Services Negotiating Committee (PSNC), prescribing pharmaceuticals represents the second-highest cost in the United Kingdom (UK), after medical staff [3]. As of 2019, around $1.25 trillion USD had been spent on medicines globally, up from only $887 billion in 2010. The spending on medicines is anticipated to increase to $1.59 billion by 2024 [4]. By 2019, the UK had around £127 billion spent in healthcare [5]. Such figures indicate an extensive waste of resources in the healthcare system. This waste includes inappropriately prescribed medication, which results in the overstocking of medications. Gebremariam et al. [6] reported that supply chain management and related variables were principal contributors to the generation of pharmaceutical waste. Likewise, poor storage conditions, storing medicines on the floor, the absence of specific stocking plans, poor climate control, and overstocking expired medicines can lead to significant medication spoilage [6]. 

The last phase of the pharmaceutical waste is disposal, traditional burning or non-burning technique utilized. It is essential to note that, out of all pharmaceutical waste, only 15% is hazardous, whilst the remaining 85% is general [7]. Large amounts of prescribed pharmaceutical waste are found in the waterways, streams and groundwater, and it has similarly been shown that a percentage of these are affecting the water and the climate [8]. The WHO classification of different types of healthcare waste is [7]:

- pathological; this includes body parts, body fluids, human waste, and tissue waste and animal corpses that are contaminated;
- pharmaceutical; this is either unused, contaminated medicine or medicine which has expired;
- cytotoxic; genotoxic waste (highly hazardous);
- sharps; includes syringes, needles, and blades, etc.;
- infectious; this usually contains blood or any bodily fluid which is contaminated and could, therefore, infect other people when they come into contact;
- non-hazardous; these waste materials can not cause any chemical, radioactive, biological, or physical dangers; and,
- radioactive; products that are infected by radionuclides.

These different types of waste require differing methods of disposal and/or new approaches in order to reduce or eliminate waste. It is important to determine the most suitable method to help preventing/reducing the negative consequences of the disposing methods on the environment, specifically on water, soil, air, and on human well-being [7,8].

The circular economy (CE) is a holistic philosophy that is conveyed through a system for managing and preserving resources ‘in use as long as possible through recovery and reuse’, hence circularity [9]. The CE approach closes the gap between production and the life cycle of the natural ecosystem upon which individuals rely for business and physical survival. It signposts practical ways of eliminating waste, transforming biodegradable and non-biodegradable waste, and promoting reuse and recycling. In CE, a distinction is made amongst different choices of circularity, represented as the R-model of 3R, 4R, or even 9R models (the 9R model being the optimal application of CE incorporating Refuse, Rethink, Reduce, Reuse, Repair, Refurbish, Remanufacture, Repurpose, Recycle, and Recover) [10,11]. Kirchherr et al. [12] claimed that the CE is ‘the combination of reduce, reuse and recycle activities’ to ensure systematic change. The CE has been rapidly growing to realise the United Nation Sustainable Development Goals (SDGs) and as an alternative strategy for business advancement. In the CE, products and services operate in closed loops (being produced and then recycled for further use) and they are intended to work in harmony with the environment. The Ellen MacArthur Foundation, which was founded in 2010 and aims to accelerate progress towards a regenerative CE [13], defines the CE as a move from a linear model of resource consumption, which pursues a take-make-dispose design, to an economy that is restorative by intention. The CE associates the supply and demand of supply chain industries in order to increase resource efficiency and help achieve sustainable production and consumption [11]. 

Sufficiency economy philosophy (SEP) is another approach that has been considered in academic circles as contributing to the sustainability agenda. SEP is defined by the United Nations [14] as “an innovative method for development that is designed for practical application over a wide range of problems and situations”. The objective of SEP is to improve planning procedures in order to ensure sustainability, manage changes in the world and utilise natural resources in a capable way while preserving nature. SEP is a sustainable development approach that was introduced by the late king of Thailand and implemented through three different components; moderation, which aims for the effective consumption of resources; reasonableness, which concerns objectively choosing the degree of products adequacy while considering the elements that are involved and the normally expected results; and, risk management, which entails adapting that is based on reasonable effects and changes that are projected by considering the likelihood of future circumstances from different viewpoints [15]. 

A key objective of the CE is to promote and facilitate greater sustainability. A sustainable supply chain is defined as a supply chain, in which operations, assets, data, and funds are managed to increase supply chain production while simultaneously reducing environmental effects and improving social wellbeing [16]. As mentioned by the European Union (EU) parliament [17], the application of CE practices for waste management in general could help to save EU organisations nearly €600 billion through, for example, waste avoidance, eco-friendly products and reuse programmes. It could also help minimise yearly greenhouse gas emissions by 4%. The benefits of the CE include improvement of the environment, improvement of the safety of raw materials, acceleration of innovation, and improvement of economic growth [18,19].

An example of the CE in practice can be seen in closed-loop supply chains, in which recyclers and manufacturers collaborate and work closely to realise resource and cost savings. Many sectors have adopted closed-loop supply chains in their processes. In the medical field, GE Motors and Philips [20,21] have started to refurbish medical products, including magnetic resonance imaging (MRI), computed tomography (CT), ultrasound, and X-ray machines, by obtaining full control to guarantee that all exchanged materials are repurposed or reused and produced with high quality to ensure the efficiency of the products. 

The aim of this study was to determine whether the application of CE principles reduce pharmaceutical waste and support sustainability in the Pharmaceutical Supply Chain (PSC). The rationale of the aim of this research was to identify ways to decrease the negative environmental impact, costs and promote sustainable supply chain and eco-design through the application of CE principles in the PSC. By identifying how the CE principles and the R-strategies can be used in the pharmaceutical supply chain it will be possible to determine how the negative impact of pharmaceutical waste be reduced in terms of costs, sustainability, and increasing circularity. To achieve this aim, the following objectives were posited: (1) to ascertain how pharmaceuticals waste is created, (2) to better understand how this waste is managed, (3) to outline how it is safely disposed, and (4) to determine how pharmaceuticals waste can be reduced and better managed through the adoption of CE principles.

## 2. Materials and Methods 

A review of current pharmaceutical waste management studies was undertaken to document how pharmaceuticals be reused and whether implementation of the CE philosophy and associated principles could help to reduce waste. The following keywords were used for the primary search: ‘Medicines’ AND ‘Pharmaceutical’ AND ‘Pharmaceutical Waste’ OR ‘Drugs’ OR ‘Pharmaceutical Return’ OR ‘Disposal’ OR ‘Hospitals’ OR ‘Pharmaceutical Supply Chain’ OR ‘Medicines Reuse’ OR ‘Circular Economy’ OR ‘Circular Economy Principles’.

First, the titles and abstracts of each article were screened, and the most significant articles were selected. Second, the related abstracts were chosen, and the full form of each selected article was retrieved. A few papers were eliminated after their selection, as described below. Journals and papers published in English were chosen. Articles, papers, and studies published before July 2020 were explored while using Elsevier, Google Scholar, MDPI, PubMed, SAGE, and Science Direct.

To be included in the review, articles/papers had to be related to pharmaceuticals, medicine reuse, waste management, and/or CE, and they had to present new and/or relevant information. Articles/papers on approaches to waste management improvement, legislation, the PSC, waste generation minimisation, and CE application were also included. Excluded papers are not explicitly relate to the keywords highlighted above.

**Figure 1:**
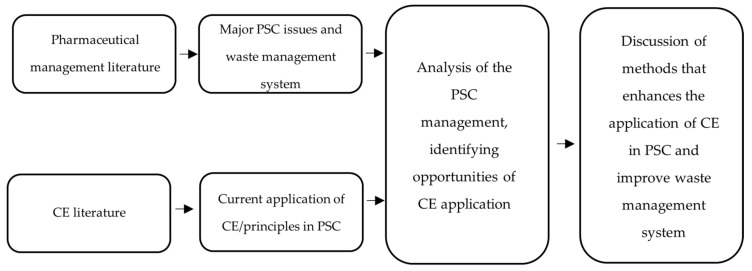
Areas of literature discovery.

The search was conducted while using electronic databases, avoiding manual exploration. Duplications were eliminated. Non-academic grey literature was also searched in Google utilising similar keywords. These sources included journalistic articles, reports, and webpages on pharmaceuticals waste and CE. A conventional quality examination was not utilised, as one of the goals of this study was to gather a broad base of proof, including all of the procedures and studies related to gathering in-depth literature data. Figure 1 shows the areas of the literature that were reviewed to meet the aim of this study.

## 3. Results

### 3.1. Pharmaceutical Waste Management

The literature review identified three clearly defined areas of focus when examining pharmaceutical waste management. These are discussed individually below.

#### 3.1.1. Waste Creation

Instances of pharmaceuticals waste may be caused by patients who are unable to utilise all of their administered pharmaceuticals due to unfavourable impacts (side effects), daily dosage modifications, health improvements, the expiry of medicines, doctors’ prescribing practices, or dispensers’ practices. Non-adherence to prescriptions can also cause stockpiling of leftover medications in the home. According to the WHO, half of the patients neglect to take medication effectively [22]. As such, families and patients around the world are in possession of unused or terminated prescriptions, and the associated dangers have prompted research interest. Many individuals who stockpile undesirable, unused, or expired pharmaceuticals in their homes dispose of them through waste containers or sinks or by flushing them down the toilet. It is important to realise that discarding unused or terminated pharmaceuticals through non-permitted methods affects the environment and individual wellbeing [6,23]. 

Table 1 shows the different waste creation of pharmaceuticals.

Unused pharmaceuticals could be the result of changes in the recommended treatment. Such practices lead to the expiration of prescriptions, which are then put away or discarded by household members who flush them down toilets instead of returning them to pharmacies [39]. Analysis indicates that £300 million worth of prescription pharmaceuticals that are authorised by the UK National Health Services (NHS) are wasted every year [41]. Such wastage accounts for a significant percentage of pharmaceutical-related expenditures in the UK. For every £25 spent on pharmaceutical products, £1 is wasted. The £300 million includes £90 million worth of unused prescriptions in people’s homes at any one time. An estimated £110 million worth of prescriptions are returned to pharmacies every year. Approximately £50 million worth of unused pharmaceuticals from care homes are disposed of every year by NHS [41].

The UK government funds its healthcare system through taxation of its citizens and businesses. Pharmaceutical waste increases the cost to the government of meeting the healthcare needs of the country. At the same time, pharmaceutical waste that results from non-adherence to prescribed medications increases the cost of treatment, because patients subsequently require additional treatment [42]. An increase in unused and expired medicine contributes to pharmaceutical waste and increases the use of financial resources [43,44,45]. Although unused pharmaceuticals have been studied extensively worldwide, there are obstacles to decreasing the number of unused pharmaceuticals. Leftover medicines in hospitals may expire and remain unused, because of a lack of proper controls [44,45]. Most hospitals experience increased pharmaceutical waste as a result of poor dispensing strategies by the pharmacy, which contributes to the overflow of pharmaceuticals [45].

Another reason for increased pharmaceutical waste is a lack of knowledge regarding medication usage and disposal [46,47]. Patients may not be educated as how best to safely dispose of their medicines. Another reason for unused medicine may be changes in treatment, meaning that the unused medications become a source of waste [46].

#### 3.1.2. Waste Management

Current pharmaceutical waste management and disposal methods and related social, economic, and environmental burdens must be understood from different points of view [48]. The lack of awareness of proper waste management in hospitals, particularly those in developing countries, has made these institutions a focal point in the spread of disease and infection, rather minimising and eliminating waste [48,49]. Research on hospitals in Kuwait found that pharmacists lacked knowledge about the consequences of sub-optimal/unsafe pharmaceutical disposal methods and often did not follow guidelines that were issued by the Ministry of Health [49]. A similar study [47] on Iraqi hospitals found that pharmacists needed programmes to improve their knowledge of appropriate disposal methods for pharmaceuticals waste. Inadequate training and a lack of awareness among hospital staff, such as nurses and pharmacists, contribute to the increase in pharmaceutical waste in many countries. Control and visibility are crucial in the reduction of losses due to expiry. When the inventory system is functioning at optimal levels, inventories could be redistributed within the system in order to enable a quick workflow. 

Johnson et al. [50] found that the inadequate segregation of waste increases costs to hospitals already under significant budget constraints. There is much misunderstanding regarding the best possible methods of medication disposal, and several countries do not have standard medication disposal requirements. For decades, there have been various reports about the presence of pharmaceuticals in groundwater, lakes, waterways and drinking water due to improper disposal [22]. These pharmaceutical disposal methods negatively affect natural ecosystems and human health.

Against this backdrop, there is a need to effectively manage waste and focus on avenues to control or decrease waste creation. Waste management practices are currently undergoing significant changes from a simplified collection and sorting procedure to a sustainable smart waste management system, as per Zhang et al. [51]. This is achieved by effectively managing and focusing on product/service system designs, resource and energy recovery and end-of-life management of currently wasted resources through initiatives, such as waste reduction practices, biological and thermal processes, and material recycling techniques.

Significant efforts are underway in order to reduce pharmaceutical waste, not just for financial reasons but also to address issues related to current pharmaceutical waste disposal methods, such as landfills [52]. Returned medications are treated as waste and disposed of or destroyed [53]. Reuse and recycling remain generally unexplored, because, under current regulations, many countries, including the UK, do not allow for unused or returned medications to be reused or to enter the PSC [54].

Better waste management can be achieved by focusing on improving the efficiency throughout the value chain in terms of the production, inventory management, usage/consumption, and performance of resources. There is also a need to change the waste management approach by introducing waste management plans that enable facilities to plan for all necessary resources, including staff training; to monitor and evaluate the waste generated from the facilities; and, to take charge of all activities that are likely to generate waste [55]. In addition, waste management also requires effective segregation of waste, which is key to reducing the volume of waste that needs attention and ensuring that each treatment process only receives compatible waste [55]. The introduction of a digital track and trace system would provide timely information to support the production and distribution of medicines in order to optimise medicine production and reduce overproduction, which leads to the generation of waste [55,56,57].

A large amount of waste is generated when there is lack of visibility of waste generation by the hospital management. Capturing data usage and using that in supply chain management is critical for reducing waste [58]. Tracking utilisation ensures that a facility only has what it needs without excesses or wastage. A balance must also be maintained between what gets produced, ordered, and distributed. Forecasting is critical in maintaining a balance in the healthcare management system. Pharmaceuticals waste is currently managed and processed in multiple ways, and all stakeholders, e.g., manufacturers, general practitioners (GPs), pharmacy, care homes, and patients, in the pharmaceuticals waste management context play roles in waste management, treatment, and disposal. 

#### 3.1.3. Waste Disposal

Studies on household pharmaceuticals in Ethiopia [59], Kuwait [60], Poland [61], Saudi Arabia [62], Qatar [63], the UK [64], and the United States [65] concluded that most unused and expired pharmaceuticals are disposed of in the garbage, as there is no clear guidance for patients on the proper disposal of medications. The causes of medication wastage are different in each household. The death of a patient, changing from one medication to another, stopping treatment and lack of consistent use by patients all contribute to pharmaceutical waste [59,60,61,62,63,64,65]. Continued pharmaceutical waste over time significantly impacts the environment [8].

The Basel Convention recommends that all healthcare organisations follow waste treatment methods that reduce the release of chemicals or hazardous waste [66]. Likewise, the WHO recommends following waste treatment methods that help to reduce the release of chemicals while recognising the differences in local conditions and availability. To a large extent, poor disposal practices are often due to an absence of adequate training for clinical staff. Insufficient hospital funding also leads to improper waste disposal [60,61,62]. Table 2 shows the advantages and disadvantages of different pharmaceuticals waste disposal methods.

Landfills and incineration are the two most utilised disposal strategies [71]; these alternatives for the final removal of waste are utilised to various extents worldwide. In every country, geographical, economic, social, technical, and other factors must be considered when selecting preferred waste disposal methods. In the EU and Japan, incineration is viewed as the preferable method of pharmaceutical waste disposal, and landfills are considered to be a last resort. 

Final safe disposal of pharmaceuticals waste is critical, given the potential public health risks that are associated with this type of waste. The most effective way to minimise and dispose of pharmaceuticals waste is to separate waste at the generation stage [72]. It is important to separate waste streams in the workplace to protect people and the environment, regardless of the disposal and treatment strategy. Separation involves sorting various types of waste while using liners with different coloured codes or original packaging in which they are produced. This has regularly been the main task of pharmaceuticals waste disposal. The lack of adequate separation of pharmaceuticals waste increases the risk of workplace accidents and blood-borne viral infections [73].

Pharmaceutical disposal methods need to be adhered to properly, and pharmaceuticals should be returned to a predetermined pharmacy controlled by the health ministry [31]. Pharmaceutical waste disposal frameworks are available in certain nations, and many countries follow the same framework in dealing with returned or expired medications. In the UK and New Zealand, individuals are advised to return expired and unused medications to the pharmacy for safe disposal and they are advised never to dispose of them down toilets [31,33]. In the United States, a system for managing pharmaceuticals waste removal has been developed under the regulations of the Food and Drug Administration (FDA) and the Environmental Protection Agency (EPA) [74]. Australia has implemented a programme (NATRUM) that accepts returned and unwanted medicines for free [75]. 

#### 3.1.4. Waste Reuse and Recycling

Current methods of disposing of unused pharmaceuticals, including expired pharmaceuticals, have become a global issue. Take-back programmes for pharmaceuticals are eco-friendly and they have been implemented in many countries, as discussed by Alnahas et al. [48]. The objective of these programmes is to safely dispose of pharmaceuticals returned by patients who no longer need them. Many countries do not permit returned medications to be re-dispensed. For example, the UK requires disposal, even if the medications are in good condition and have not been used. However, a study of pharmaceutical reuse in the UK concluded that, based on the findings of the interviews data, reusing unused medicines would reduce NHS spending and lower manufacturing costs [46]. 

The United States and Greece allow for medications to be reused to make medicine more affordable for people who would not otherwise be able to pay for them [76]. These programmes involve the collection and reintroduction of medicines to the original processing location to be recycled or reused by the government, retailers, or manufacturers. For instance, SMARXT Disposal, a partnership between the American Pharmacists Association, Pharmaceutical Research and Manufacturers of America and Fish and Wildlife services, has developed awareness campaigns for take-back initiatives that involve a collaborative structure for pharmaceuticals waste reuse [77]. This programme helps to reduce the cost of pharmaceuticals and improve the efficiency of the supply chain. Similar programmes have been initiated around the world: in Canada, the ENVIRx programme accepts unused and expired medication for proper disposal; New Zealand adopted the Disposal of Unwanted Medicines Properly (DUMP) programme to encourage individuals to return unused and expired pharmaceuticals; and, in Australia, the Return Unwanted Medicines (RUM) programme was adopted for proper disposal [78,79].

When medicines are returned to physicians, they are destroyed according to guidelines that operate on the assumption that these end-of-life items are useless, as mentioned by Breen [80]. This process could be improved by collecting information about the returned products from GPs, pharmacists, administration improvement managers, and commissioners. Information, such as the amount prescribed, to whom it was prescribed and when it was administered, could be utilised in order to improve prescribing, minimise waste and improve medicine optimisation [80].

Open medicines return events can be jointly held by pharmacies and local councils to help spread awareness in communities about the importance of returning medicines so that they can be destroyed according to government guidelines. These events can help to minimise pharmaceutical waste and increase recycling and the proper disposal of medication [81]. They can also help to increase awareness regarding how following appropriate procedures helps minimise environmental effects [81]. Raja et al. [82] proposed that governments develop and implement a national medication return programme to gather unused or terminated medications at each hospital, so that they can be disposed of properly. Improving the pharmaceutical waste management system and achieving a green PSC requires the cooperation of the entire supply chain from the manufacturers and wholesale suppliers to the GPs, community pharmacies, and patients. 

Research that was conducted by Hsieh et al. [83] indicates that the lifespan of specific Active Pharmaceutical Ingredients (API) can be extended where APIs from medicines can be recovered and reused for new formulation development if they do not contain excipients. The recovery process can be done using green engineering, a technique that uses water for the separation process and mechanical energy to provide the power for membrane separation. The process used are tablet milling and dissolution, solid-liquid separation, diafiltration by ultrafiltration, reverse osmosis membrane operation, and crystallization. The recovery process helps to reuse of the API and minimize the cost of API production [83]. Some important points need to be considered for the recovery process [84]. The purity of the reused API should be close to that of a new API, which includes, for example, its density and flowability, while the concentration of the polymer that is recovered should be insignificant, making sure that chemical degradation is avoided by using a suitable temperature during the process, and avoiding any harmful chemicals in order to ensure a low-cost green process [84]. The API recovery cycle is considered to be green because any solvents from waste are completely recovered and reused.

#### 3.1.5. Obstacles to the Safety and Quality of Returned Pharmaceuticals

Regulatory agencies must have strict quality control and safety monitoring measures in order to affirm the appropriateness of medications for reuse. Such procedures include monitoring by specialists to confirm the capacity and limit any risk of damage, contamination, or infection. Moreover, the proper reuse and recycling of medications can reduce the environmental impacts of improper or illegal disposal of pharmaceuticals and reduce the associated carbon footprint.

Table 3 identifies some barriers to the safety and quality of returned medications, which may affect redistribution. The safe disposal of medicines determines the standards of quality in the PSC. Health and safety are essential factors to consider in the PSC in order to protect consumers from infections, complications, and side effects from medications, as well as death due to improper medication usage.

The decision to reuse returned pharmaceuticals depends on a safety confirmation process, whereby devoted analysers at pharmacies can process unopened, intact, and authentic pharmaceuticals. Using technology and engaging arranged networks in smart pharmaceutical packaging will help to determine whether returned, unused, and unexpired medications are safe for reuse [57].

Huge strides have been made in the design of secure pharmaceutical packaging in ensuring trust and confidence in the integrity of medication [2,86,87,88]. This advances further support the global Falsified Medicines Directive launched in 2019, which ensures product integrity within the supply chain from the point of production through to customer sales [89]. Product protection within the supply chain was reinforced in 2018 when the International Organization for Standardization (ISO) published the new ISO standard 21976:2018, entitled "Packaging-Tamper verification features for medicinal product packaging", and again in 2019, when pharmaceutical companies that have prescription medicine in their portfolio were required in order to provide additional security features in accordance with the Anti-Counterfeiting Directive 2011/62/EU [90]. Stakeholders within the pharmaceutical supply chain need to know that medication has not been tampered with or affected by transportation/storage conditions. These developments in policy, along with high-tech tamper proof solutions and innovative pharmaceutical packaging that provides patients with clear instructions, prevents harm to the environment and conforms to government guidelines and strategies, support the premise that medication could be reused [2,91,92].

### 3.2. Circular Economy and the Management of Pharmaceutical Waste

When considering the application of CE principles to the PSC, the best methods for reducing pharmaceutical waste include reducing, reusing, and recycling disposable instruments and materials. CE offers several advantages to healthcare services, including cost savings, high quality of life, and continual service improvement [93]. Pachauri et al. [94] found that the CE promotes the use of sustainable products by replacing nonbiodegradable raw materials. The entire operational process is interlinked to accomplish sustainability, as the waste and products of one phase become the raw materials for other products or procedures. 

As stated earlier in this discussion, the basic principles of CE are the three Rs; Reduce, Reuse, and Recycle. Examples of the application of these principles can be seen within the PSC. However, the more advanced principles of CE extend past the 3Rs and they present a stronger proposition, which are the 9Rs (see Table 4) [95]. Each R prompts product owners to focus on their creation, use, and disposal, in order to expend their lifespan, but where feasible also maximise the use of materials. This, is turn, contributes to the United Nations Sustainable Development Goals, to end poverty, protect the planet and ensure that all people enjoy peace and prosperity by 2030 [96]. 

There are a number of excellent examples in the pharmaceutical supply chain where innovative practices are in place in order to promote the reduction of waste creation (reduce), to enhance the reuse of medication where legal and possible (reuse), and recycle products or product components where legal and feasible (recycle). These clearly support the CE ethos. Table 5 illustrate some examples of the practices.

At present, the used substances are considered to be obsolete, infectious, and harmful to society and the environment [105]. However, easing strict guidelines in pharmaceutical reuse could pave the way for strengthening circular principles in the healthcare economy [105]. Connelly [76] argued that this could also help pharmacists re-dispense returned medicine to other patients who need treatment, helping to reduce pharmaceutical waste in health facilities. 

Recently, in response to the COVID-19 pandemic, the NHS in the UK released guidance for reusing medications in assisted care homes and hospices [106]. The government choice to reuse pharmaceuticals under strict governing criteria is an attempt to manage medical deficiencies and shortages during this period only. All of the prescriptions not required by the individual for whom they were initially prescribed can be reused under the management of registered healthcare professionals and proper recordkeeping [32]. However, some patients may not accept returned medication, citing concerns regarding the proper storage of unused medicines, e.g., room temperature, humidity or sanitation [8]. The reuse of medications to treat different patients during pandemic is especially feasible in cases where patients no longer need the drug, e.g., if they have died, or the provision of their medicine has been interrupted. Reuse applies to all medications—including fluid prescriptions, injections (analgesics, insulin), creams, and inhalers—that are in sealed or closed packs [9,32].

When contemplating the adoption of the CE into the PSC, key factors that should be considered are presented in the Political, Economic, Social, Technological, Legal, and Environmental (PESTLE) model, which supports or challenges practice change. The following PESTLE analysis is proposed based on the literature reviewed in this study (Table 6).

In CE implementation, a definitive objective is to hold the essential value of items using an item for as long as might be feasible and in a closed loop, such as reuse and recycle. The most suitable path must be explored in terms of approaches and legislation in order to set up circular material dissemination in a closed circle and ensure the sufficiency of the production and consumption of pharmaceuticals. Progress towards CE implementation requires interactions between specialists, the legislation, the supply chain, production frameworks, and utilisation, which are controlled and characterised through authoritative, financial, and instructive instruments. 

## 4. Discussion

It is clear from the literature presented above that there is a discerning focus on pharmaceutical waste management across multiple channels; however, what is not evident is a binding mechanism, a home for the disparate initiatives and practices to come together and make sense for the users to adopt when tackling pharmaceutical waste. We posit that the Circular Economy philosophy offers this. 

Officially, pharmaceutical waste management is an exceptionally specialised field that must be managed by qualified, skilled, and experienced staff at the administrative and ground levels. A large amount of waste is generated in healthcare institutions, due to a lack of proper systems, inadequate training, and a lack of balance between supply and demand within the healthcare inventory management system, which can increase financial and environmental issues. Proper measures should be taken, especially medicinal stock management, evaluation, quantification, procurement, and utilisation, in order to improve the supply chain and minimise waste. There are many examples of excellent practice that aspire to reduce, reuse, and recycle medicines, as noted in this study, but more needs to be done to maximise these efforts and offer clear steering on standardised methods that can be adopted and built into pharmacy practice. We see clear reference in this work to the basic 3Rs of CE, Reduce, Reuse, and Recycle, but endorsing CE in its entirety would prompt pharmacy, pharma, and healthcare professionals to consider the use of green product design, production, logistics, and dispensing to patients to move closer to the optimal 9Rs of CE.

Pharmaceutical waste management can also be both exacerbated and more effectively managed by the actions of healthcare providers and patients by inappropriate prescribing, repeat medication requests, lack of compliance to medication regimes, and stockpiling, as highlighted earlier in the paper. Measures can be taken in order to identify sources of waste creation and take steps to address these to maximise product utilisation when in the system as a finished product, but also reduce additional risk to the patient, their families in their homes (by returning unwanted stock to pharmacies), and also the environment (using safe disposal methods). The role of the patient in supporting effective pharmaceutical waste management should not be underestimated. 

From a systemic approach, the adoption of the CE and associated principles can support waste reduction across the entire pharmaceutical supply chain. While reusing and recycling medicine helps to reduce pharmaceutical waste from environmental and economic perspectives, their application needs further improvement and approval by economic and government authorities and endorsement from all supply chain stakeholders. Moreover, it is important to redesign the current pharmaceutical product life cycle to facilitate medication reuse and minimise waste. 

**Figure 2:**
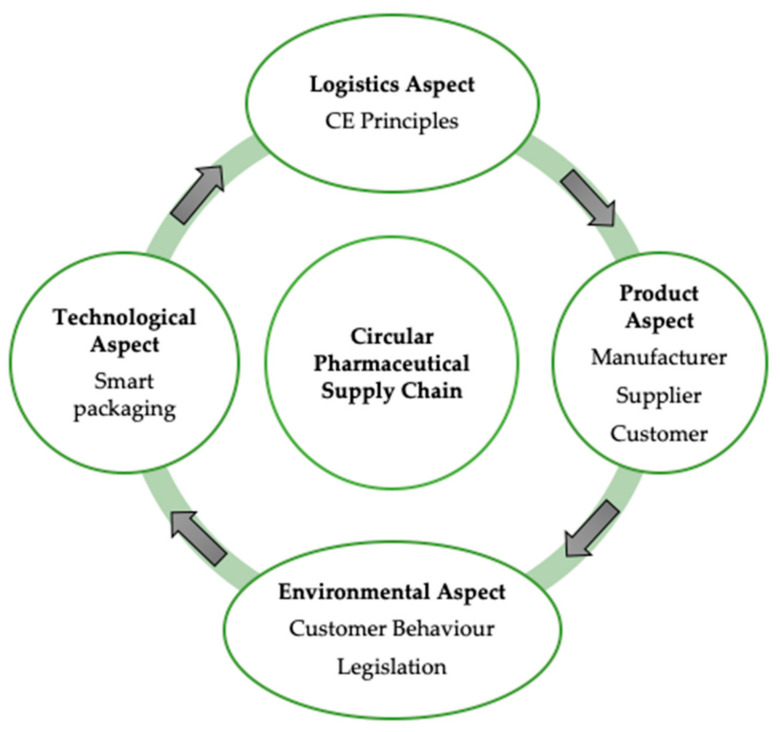
Aspects of the Circular Pharmaceutical Supply Chain (CPSC).

Four key aspects of creating a Circular PSC (CPSC) related to the internal mechanisms of associations and the duties of various PSC actors have been identified. Figure 2 shows how these aspects are interconnected and how they affect the implementation of the CPSC.

A set of legislative guidelines regarding the reuse of medicines must be set in order to limit wastage and introduce CE principles throughout the PSC, endorsed by all stakeholders. By resolving conflicting goals and involving all stakeholders, including packaging manufacturers, recyclers, decision makers, society, and consumers, pharmacists can legitimately and supportively influence patients’ understanding of proper medicine use and commitment to reducing waste generation through proper use and disposal. They can also educate their patients on medicine-related issues, such as proper medicine use, pharmaceuticals waste, and appropriate disposal and return methods for unused and expired medications.

Thus, the use of the CE as a binding mechanism for existing and potential waste reduction practices can improve pharmaceutical waste management. Table 6 shows the changes needed related to moving from a linear chain (take, make, dispose) to a circular (proposed practice) PSC under an analytical level. Table 7 shows a systemic multi-level appraisal of practice changes that can be undertaken to move from a linear chain (take, make, dispose) to a circular PSC (proposed practice to expand product lifespan and material reuse).

**Figure 3:**
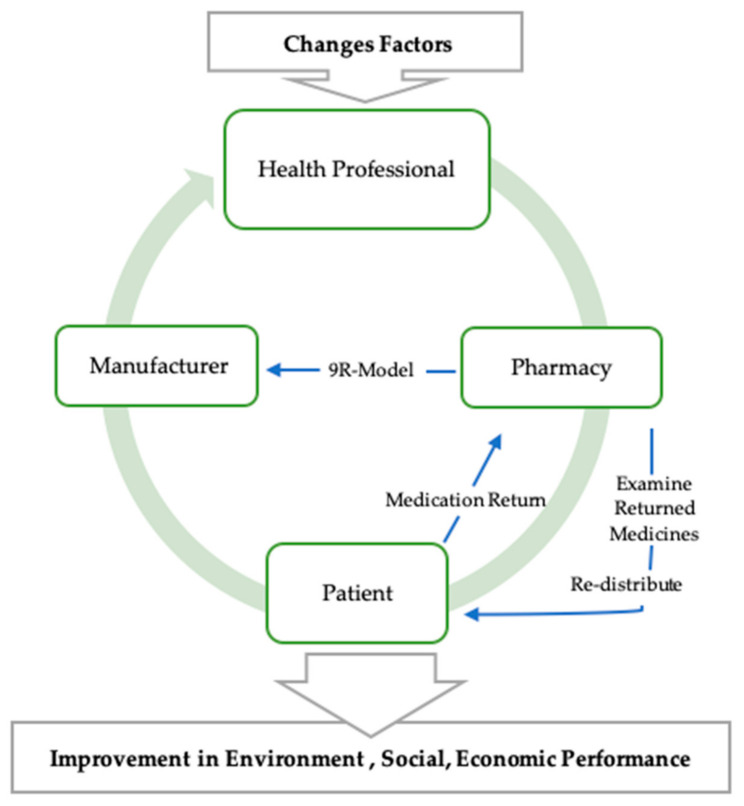
Circular Pharmaceutical Supply Chain (CPSC).

The proposition is based on moving from the linear traditional approach of making products, use, and dispose, to (1) designing for potential future re-use (having components that can be remanufactured/reconfigured to be part of a new product e.g., inhaler cartridges); (2) making only what is needed; (3) using in a thrifty manner (both support SEP philosophy); (4) reusing products/components where possible (e.g., API extraction); (5) disposing in an environmentally safe capacity; and, (6) offering growth capacity in order to promote greener practices and a 9R agenda. Figure 3 shows the changes factors needed for CPSC.

The limitations of this study are that it is theoretical and as such the conceptual model developed does need to be considered further and tested. Further research would be to undertake qualitative analysis with stakeholders in the PSC in order to determine their views on current waste management practices and the application of CE principles to the PSC and the adoption of CPSC.

## 5. Conclusions

This study aimed to determine whether the application of circular economy principles can minimise pharmaceuticals waste and support sustainability in the pharmaceutical supply chain. The results show that there are a multitude of practices that are used in order to reduce pharmaceuticals waste, but these are not always targeted or informed by any guiding principles or strategies. The CPSC as designed advocates that all stakeholders in the supply chain should contribute to effective management of medicines which includes design, production, use, reuse, and recycling (at its optimal level incorporating 9R practices). The following conclusions are proposed. 

In our exploration of the application of CE to the PSC and the conceptual model produced, the CPSC, we acknowledge that there are existing examples of CE in this supply chain, but these reflect the application of the 3Rs as opposed to the wider remit of CE, the 9Rs. How the 9Rs can be applied throughout the PSC is unclear at present. 

The success of the embodiment of the CPSC relies on both government and professional body endorsement and the recognition of pharmaceuticals waste management in pharmacist training and development. It also requires the inclusion of skills from other disciplines, e.g., engineering and management (for product/technology/systems development), in order to ensure greener thinking from product conception through to use and safe disposal by the patients.

## Figures and Tables

**Table 1:** Waste creation point and issues associated.

Waste Creation Point	Issue	Current Resolution/Practice
**Manufacturing**	Overproduction of stock based on forecasted demand.	Secure accurate demand based on transparency and sharing of information across the supply chain facilitated by government bodies [24,25]
	Overproduction of stock based on actual demand, e.g., a medicines shortage (but short lived so excess stock is created).	Ensure the transparency of stock production and use and effective reporting of medicines shortages between pharmacies, wholesalers, and manufacturers [24] Distinguish the cause of the shortage and focus efforts there to increase or use on-hand supply [24,25]
**Pharmacy**	Overordering of stock by pharmacy.	Implement effective procurement training and inventory management systems [26]
	Insufficient storage conditions by pharmacy.	Conduct regular checks ensure suitable conditions of light, humidity, ventilation, temperature, and security [26]
**Hospital Wards/Clinics/Estates**	Excess stock requested and held by wards or clinics.	Create stock lists at the ward level with the support of pharmacy store teams to manage stock levels of wards effectively [27]
	Incorrect medication prescribed for patient and not enough or unclear information given.	Enact effective processes to process and dispense prescriptions supported by accurate information from a consultant to avoid irrational medication. Also, ensure that clarification is offered to the patients regarding the dosage, use, and advantages and disadvantages of the recommended pharmaceutical [28,29,30]
	Patient is deceased but medication is in their name and cannot be used by anyone else.	Reuse prescribed medications if the patient is deceased. This applies if, for example, there is no available stock, no available alternatives, and there is no risk associated with other patients using the medicines [31,32]
	Medicines not rotated or used effectively (manual intervention based on expiry dates) or inventory management systems not utilized effectively to reduce stock obsolescence.	Provide effective training for staff and use of inventory management systems [26]
	Patient’s own medicine lost on admission and, therefore, are not available for use.	Encourage patients to bring their own medicines. Design system to ensure patients’ own medicines stay with them using green bags, e.g., the green bag scheme for improving the utilization of prescriptions for better results and decreased waste [29,30]
	Inadequate resources to support effective management of pharmaceuticals waste segregation and disposal.	Create dedicated resources to support pharmaceuticals waste management and safe disposal [33] Both small-scale (e.g., training programs) and large-scale (e.g., legislative and administrative) solutions are needed to ensure safe waste management [34]
**General Practitioner (GP)/Consultants**	Overprescribing by GPs/consultants.	Undertake informed prescribing in relation to quantity and frequency, guided by current data on stock availability provided by government bodies [35,36] Develop a system to permit patients to improve their overstocking and ordering of medication [35,36]
	Remote prescribing by GPs.	Remote prescribing are applied care home. But is being addressed with the introduction of pharmacists to manage prescriptions more effectively [37,38,39]
**Care Homes**	Excess stock received and held for patients.	Educate staff to contact GP regarding prescribing patterns and use a pharmacist to support medicines use [31,32,33,34,35,36,37,38,39,40]
**Patients**	Repeating prescriptions requested by patients.	Educate and facilitate patients to request stock when needed and approved by GP without overstocking [34,35,36,40]
	Advising GP or healthcare professional when they cannot take medicines and no longer needed.	Educate and facilitate medicines returns to pharmacy, GP, or another reliable repository [38]

**Table 2:** Medicines waste disposal methods.

Treatment	Advantages	Disadvantages
**Incineration** [67]	Low cost, accepts different waste types, minimises the waste volume	Not environmentally friendly, increases pollution, high cost
**Autoclaving** [68]	Environmentally friendly, used for infectious waste and sharps	Does not minimise the volume and is not cost effective
**Microwave Irradiation** [69]	No combustion or gasification, minimal emissions	Not applicable for all waste, high cost
**Pyrolysis** [69]	Environmentally friendly, disposes of all kinds of waste, minimises the waste volume	High cost, requires certified professional workers
**Landfill** [68]	Low cost	Not environmentally friendly, increases health risks
**Recycling** [70]	Environmentally friendly, reduces cost	Not all types of waste can be recycled

**Table 3:** Obstacles to the safety and quality of returned pharmaceuticals.

Issues	Obstacles
**Safety** [2,31,76,85]	Returned medicines may have been subject to intentional tampering, e.g., incorrect packaging. Some presently utilised seals on external medication packaging lack careful designs and effectiveness. Packaging may be unsealed. Packaging may have been contaminated while in a patient’s possession.
**Quality** [2,31,76,85]	Medicines may have been stored in undesirable conditions, e.g., temperature, moisture, light. Medicines may have an undesirable smell. Counterfeit medicines via a redistribution scheme. The dispensing and expiration dates may affect the quality of the medication.

**Table 4:** 9Rs Circularity strategies [95] (adapted by author).

		R-Strategies	Aim
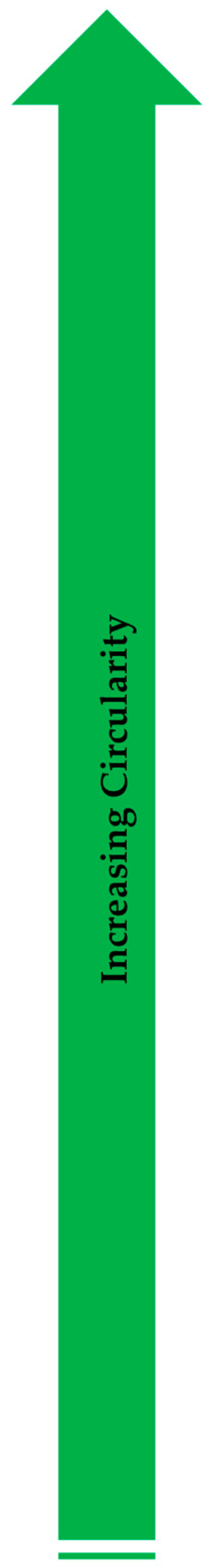	**Better Use of Products and Manufacture**	**(0) Refuse**	Make product redundant by abandoning its function or by offering the same function with a radically different product.
		**(1) Rethink**	Make product use more intensive (e.g., by sharing product).
		**(2) Reduce**	Increase efficiency in product manufacture or use by consuming fewer natural resources and materials.
	**Expanding the Lifecycle of Product and Elements**	**(3) Reuse**	Reuse by another consumer of discarded product which is still in good condition and fulfils its original function.
		**(4) Repair**	Repair and maintenance of defective product so it can be used with its original function.
		**(5) Refurbish**	Restore an old product and bring it up to date.
		**(6) Remanufacture**	Use parts of discarded product in a new product with the same function.
	**Useful Application of Material**	**(7) Repurpose**	Use discarded product or its parts in a new product with a different function.
		**(8) Recycle**	Process materials to obtain the same (high grade) or lower (low grade) quality.
		**(9) Recover**	Incineration of material with energy recovery.

**Table 5:** Examples of current pharmacy medicines waste management practice that endorses the circular economy (CE) ethos.

Product/Practice	Action
**Drug Donations** [9,94,95,97,98]	Consideration of how medicine donations from medicinal services and patients could help reduce waste and increase the reuse and recycling of medicines. These medicines could be used for individuals who cannot afford their medication.
**Epinephrine Injection** **(EpiPen)** [99,100]	Extension of the product’s lifecycle, as prompted by medicine shortages.
**Falsified Medicines Directive (FMD) and Support of Anti Counterfeit Technologies** [101]	The introduction of FMD and the adoption of technologies to reduce counterfeit drug presence in the supply chain. This increases the transparency of stock, increases confidence in stock integrity and reduces risk of patient harm.
**Inhalers** [102,103]	Promotion of more environmentally friendly inhalers and recycling of outer packaging/cartridges for reuse.
**Medication Dosing—Cancer Treatments** [31]	Based on group volume, offering clinics to share vials of medications, ensuring maximum utilisation of stock and reducing waste and cost. This also positively impacts stock creation and holding within the supply chain due to the reduced risk of obsolescence.
**Prescribing (quantity/frequency)** [26,41,48,52]	Consideration of a practice of prescribing medicines in specific quantities and frequencies, which can smooth out the demand for specific medications, reducing the risk of shortage and domestic stockpiling. This practice could also promote the equity of access to medication.
**Return Schemesfor Medication Reuse** [46,104]	Verifying the safety and quality of returned medications and ensuring that medicines have tamper evident packaging to help endorse the reuse of medication scheme.
**Return Schemesfor Safe Disposal** [79]	For example, DUMP schemes, where patients are encouraged to remove unwanted products from their homes to reduce risks to patient and family safety and reduce potential environmental harm.
**API Recovery for Reuse**	Green engineering technology to recover and reuse the API (extracting, purifying, and repacking) can help to minimize waste and provide it value again [48,83].

**Table 6:** Political, Economic, Social, Technological, Legal, and Environmental (PESTLE) analysis of the adoption of the Circular Economy in the Pharmaceutical Supply Chain (PSC).

Criteria PESTLE	Enablers	Barriers	Importance
**(P)** [107,108,109]	Tax incentives in the PSC positively support the CE. International agreements and collaborations lead to the enforcement of effective policies in the PSC. Government funding exists for CE projects.	There is inadequate government funding to shift the PSC to the CE. There is no effective enforcement strategy to shift the pharmaceutical sector to the CE. The discriminatory implementation and establishment of PSC policies discourage the shift to the CE.	Political factors help to set the directions and encourage innovation through funding.
**(E)** [80,81,82]	Reduction in the use of pharmaceuticals leads to minimised costs. An increase in household healthcare expenditures creates a need to reduce the cost of production.	There are pricing pressure in the PSC between different suppliers. The high cost of establishing the CE is challenged by the low revenues of the pharmaceutical industry. The current medicine taxation system is a barrier to the transition to the CE.	The economy is a significant determinant of the running of the PSC because it guides supply and demand.
**(S)** [46,109,110]	New preferences in the population regarding the form of medications that are administered enable the shift towards the CE. Suppliers in the sector have a shared CE vision, which facilitates achieving it.	There is resistance from internal PSC and society to change from linear production to the CE in the PSC. Insufficient information on the recycling and reuse of medicine and related benefits results in hesitation to change. There is an absence of technical skills in applying the CE in the PSC while saving on costs.	Social factors determine the demand for medications by consumers of pharmaceutical products and their medication disposal behaviours.
**(T)** [2,92,110]	Secure information sharing systems are needed within medication tracking systems. Technology makes it easier to engage with patients. Technology helps minimise the stockpile of medications on the shelf and efficiently manage the stock. Advanced medicine manufacturing technology supports the transition to CE.	The cost of developing and applying a new advanced technology to transition to the CE is high. There is inadequate expertise in running the technical equipment needed for the CE in the PSC.	The PSC relies on technology for production and efficiency in its operations.
**(L)** [82,111,112]	Proposals concerning the reduction of waste produced by pharmaceutical manufacturers support the CE. Regulations on standards of pharmaceutical distribution process support the CE.	There is a lack of systems to measure and assess the CE in the PSC. There is a lack of effective legislation on poor waste management. The existing laws are not clear about pharmaceutical producers’ responsibility for waste management.	The PSC requires legal regulations to guide supply chain operations.
**(E)** [62,63,64]	Pharmaceutical and biotech companies’ high levels of energy consumption drive them to seek more eco-friendly means of operation. Emphasis that is placed on the benefits of recycling, reusing and reducing medicines supports the production of medicines in a CE. Needs to change the poor management of pharmaceutical disposal methods.	There is a lack of adequately set strategies on the recycling and reuse of medicine in an environmentally friendly way. Existing laws are not clear about the responsibility of the producers regarding waste management. Individuals’ awareness of proper medicine disposal is low.	The PSC is a sector that must meet high standards of quality, which are achieved by improving existing environmental conditions.

**Table 7:** Proposed changes needed to move toward a Circular PSC.

Level of Analysis	Focus Area	Consideration for Change
**Meta Level (Global)** E.g., WHO, UN, International Federation of Pharmacists	Recognition and endorsement Policy generation	Global recognition of pharmaceutical waste levels. Inclusion of waste reduction targets in global sustainability policy
**Macro Level (National)** E.g., government, suppliers/wholesalers, healthcare bodies, pharma advisory bodies	Recognition and endorsement Resource allocation CE philosophy acknowledgment Financial support Adoption of innovative technologies Awareness and education	Endorsement of CE agenda in the pharmaceutical supply chain. Agreement to provide resources to target pharmaceuticals waste reduction Acknowledgment of the value of CE philosophy in the design of waste reduction policies and practices Financial support for innovative technologies to deliver green design/logistics Building awareness of the 3Rs of CE (reduce, reuse, recycle) into pharmacist education Design and delivery of awareness campaigns to healthcare professionals and patients (co-designed output)
**Meso Level (Organisational)** E.g., hospitals, community pharmacies, GP, healthcare professionals	System design and delivery Patient education delivery and support Facilitation of medicine returns/design of collection channels Resource allocation Champion identification Strategic organisational approach to waste reduction	Creation of efficient medicine management systems to minimize waste creation and ensure safe disposal Engage in patient education to raise awareness of medicine use and waste creation channels Design effective channels for medicine returns/collections Dedicate time for medicine management training regarding ordering, storage, reuse/recycling Build teams to facilitate medicine stock management/retrieval from wards, conduct returns audits and safe disposal Identify champions to support pharmaceuticals waste reduction practices
**Micro Level (Individual)** E.g., pharmacy staff, manufacturers, suppliers wholesaler, distributors, patients	Awareness of pharmaceuticals waste creation Awareness of medicine returns Facilitation of returns Civic responsibility Engagement in educational campaigns	Engage in an educational campaign on the scale of pharmaceuticals waste and the financial, social and environmental repercussions associated with poor medicine management Adopt a personal responsibility to reduce pharmaceuticals waste as part of civic duty Work with stakeholders to design simple mechanisms to prompt medicine returns (e.g., text messages, flyers).
